# Three Different Strategies for Repair of Symptomatic or Aneurysmatic Aberrant Right Subclavian Arteries

**DOI:** 10.21470/1678-9741-2021-0439

**Published:** 2022

**Authors:** İsmail Selçuk, Hüseyin Sicim, Ümmühan Nehir Selçuk, Bülent Barış Güven, Ahmet Turan Yılmaz

**Affiliations:** 1 Department of Cardiovascular Surgery, İstanbul Sultan 2. Abdülhamid Han Training and Research Hospital, İstanbul, Turkey.; 2 Department of Cardiovascular Surgery, Kırklareli Training and Research Hospital, Kırklareli, Turkey.; 3 Department of Cardiovascular Surgery, Dr Siyami Ersek Thoracic and Cardiovascular Surgery Training and Research Hospital, Istanbul, Turkey.; 4 Department of Anesthesiology, İstanbul Sultan 2. Abdülhamid Han Training and Research Hospital, İstanbul, Turkey.

**Keywords:** Subclavian Artery, Dysphagia, Aneurysm, Brachial Plexus, Chimera

## Abstract

**Introduction:**

In this study, we aimed to present three different methods for symptomatic aberrant right subclavian artery (ARSA) surgery.

**Methods:**

We identified 11 consecutive adult patients undergoing symptomatic and/or aneurysmal ARSA repair between January 2016 and December 2020. Symptoms were dysphagia (n=8) and dyspnea + dysphagia (n=3). Six patients had aneurysm formation of the ARSA (mean diameter of 4.2 cm [range 2.8 - 6.3]). All data were analyzed retrospectively.

**Results:**

Median age of the patients (7 females/4 males) was 55 years (range 49 - 62). The first four patients (36.4%) underwent hybrid repair using thoracic endovascular aortic repair (TEVAR) and bilateral carotid-subclavian artery bypass (CScBp). Three patients (27.2%) were treated by open ARSA resection/ligation with left mini posterolateral thoracotomy (LMPLT) and right CScBp. And the last four patients (36.4%) underwent ARSA resection/ligation with LMPLT and ascending aorta-right subclavian artery bypass with upper mini sternotomy (UMS). Two of the four patients who underwent TEVAR + bilateral CScBp had continuing dysphagia cause of persistent esophageal compression. Brachial plexus injury developed in one of three patients who underwent LMPLT + right CScBp. Pleural effusion treated with thoracentesis alone was observed in one of four patients who underwent UMS + LMPLT.

**Conclusion:**

Among the symptomatic and/or aneurysmal ARSA treatment approaches, surgical and hybrid methods are used. There is still no consensus on how to manage these patients. In our study, we recommend the UMS + LMPLT method, since the risk of complications with anatomical bypass is less, and we have more successful surgical results.

**Table:** 

Abbreviations, Acronyms & Symbols			
ARSA	= Aberrant right subclavian artery	HL	= Hyperlipidemia
AsScBp	= Ascending aorta-subclavian artery bypass	HT	= Hypertension
CoE	= Compression of esophagus	KD	= Kommerell’s diverticulum
CoET	= Compression of esophagus and trachea	LMPLT	= Left mini posterolateral thoracotomy
CScBp	= Carotid-subclavian artery bypass	TEVAR	= Thoracic endovascular aortic repair
CTA	= Computed tomography angiography	TOAR	= Thoracic open aortic repair
DM	= Diabetes mellitus	UMS	= Upper mini sternotomy

## INTRODUCTION

Aberrant right subclavian artery (ARSA) is a rare anatomical variation of the origin of the right subclavian artery, and it occurs in about 0.7% of the general population^[1]^. It is thought to occur in embryonic life due to the insufficiency of the inhibition mechanism during the development of the aortic arch. Most of the patients are asymptomatic throughout their lives, and the diagnosis of ARSA is made incidentally by imaging methods in most of them. As a result of the compression of the aberrant artery on the surrounding tissues, symptoms such as dysphagia, cough, and stridor may occur^[2]^. Dysphagia lusoria is an abnormal condition characterized by difficulty in swallowing caused by an ARSA. It was discovered by David Bayford in 1761 and first reported in a paper by him in 1787^[3]^. ARSA may also be associated with Kommerell’s diverticulum (KD). This diverticulum is defined as the dilation of the proximal part of the aberrant subclavian artery near its exit from the aorta and represents the embryonic residue of the dorsal aorta^[4]^. Aneurysmal degeneration of KD and ARSA may result in a significant risk for dissection and rupture with high mortality^[5]^. While ARSA treatment has traditionally been performed with open surgery to relieve symptoms or prevent complications, in recent years, treatment strategies have shifted to more hybrid or endovascular approaches^[6]^. Treatment is indicated for relief of symptoms and prevention of serious complications from aneurysmal dilatation.

Open vascular surgical procedures are associated with high mortality and morbidity rates, especially in elderly patients who develop symptoms and aneurysm, and are associated with a high risk of neurological events^[7]^. In addition to open vascular surgery methods, endovascular and combined hybrid methods are applied as different techniques in order to reduce the complications that may develop postoperatively and to obtain better results^[8]^.

With this study, we aimed to contribute to the determination of the most accurate strategy in terms of results by presenting three different surgical treatment strategies in patients with ARSA and/or aneurysm.

## METHODS

Between January 2016 and December 2020, symptomatic ARSA was diagnosed in 11 patients. The data of all patients were analyzed retrospectively. Demographic data, current medical conditions, symptoms, radiological images, treatment techniques, and postoperative results of the patients were recorded (Table 1). In all patients, the location of the aberrant artery was confirmed by contrast-enhanced tomography for the analysis of anatomical structures. All patients were evaluated with computed tomography angiography (CTA).

**Table 1:** Individual patient characteristics, presentation, and outcomes.

Patient (sex/age)	Comorbidities	Pathology	Symptoms	Supra-aortic correction	Aortic approach	Complications	Outcomes
1) Male (57)	None	Aneurysmatic ARSA with CoET	Dysphagia + dyspnea	Bilateral CScBp	TEVAR	Continuing dysphagia cause of persisting esophageal compression	Partial symptom relief after esophageal dilatation
2) Female (53)	None	Aneurysmatic ARSA with CoE	Dysphagia	Bilateral CScBp	TEVAR	Continuing dysphagia cause of persisting esophageal compression	Patient disapproved additional procedure
3) Male (62)	DM, HT	Aneurysmatic ARSA with CoE	Dysphagia	Bilateral CScBp	TEVAR	None	Symptom free
4) Female (51)	None	ARSA	Dysphagia	Bilateral CScBp	TEVAR	None	Symptom free
5) Male (52)	None	Aneurysmatic ARSA with CoE	Dysphagia	Right CScBp	TOAR (LMPLT)	Plexus brachialis injury	Right upper extremity weakness
6) Female (53)	DM, HL	ARSA with CoE	Dysphagia	Right CScBp	TOAR (LMPLT)	None	Symptom free
7) Male (61)	Smoker	ARSA	Dysphagia	Right CScBp	TOAR (LMPLT)	None	Symptom free
8) Female (54)	None	Aneurysmatic ARSA with CoET	Dysphagia + dyspnea	AsScBp	TOAR (UMS + LMPLT)	Right pleural effusion (treated with thoracentesis)	Symptom free
9) Female (58)	Smoker	Aneurysmatic ARSA with CoET	Dysphagia + dyspnea	AsScBp	TOAR (UMS + LMPLT)	Recurrent laryngeal nerve paralysis	Soft hoarseness
10) Female (55)	HL, HT	ARSA with CoE	Dysphagia	AsScBp	TOAR (UMS + LMPLT)	None	Symptom free
11) Female (49)	None	ARSA	Dysphagia	AsScBp	TOAR (UMS + LMPLT)	None	Symptom free

ARSA=aberrant right subclavian artery; AsScBp=ascending aorta-subclavian artery bypass; CoE=compression of esophagus; CoET=compression of esophagus and trachea; CScBp=carotid-subclavian artery bypass; DM=diabetes mellitus; HL=hyperlipidemia; HT=hypertension; LMPLT=left mini posterolateral thoracotomy; TEVAR=thoracic endovascular aortic repair; TOAR=thoracic open aortic repair; UMS=upper mini sternotomy

**Fig. 1:**
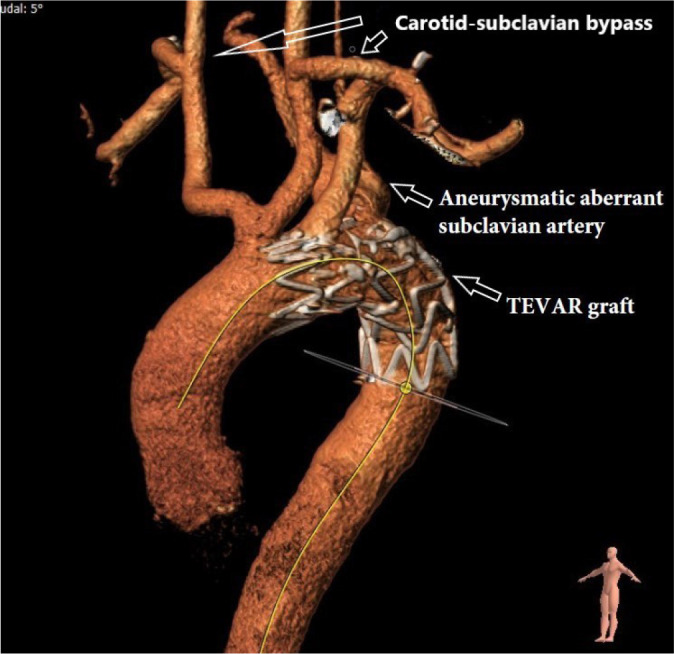
Postoperative control three-dimensional reconstructed computed tomography angiography of thoracic endovascular aortic repair (TEVAR) and bilateral carotid-subclavian artery bypass.

**Fig. 2:**
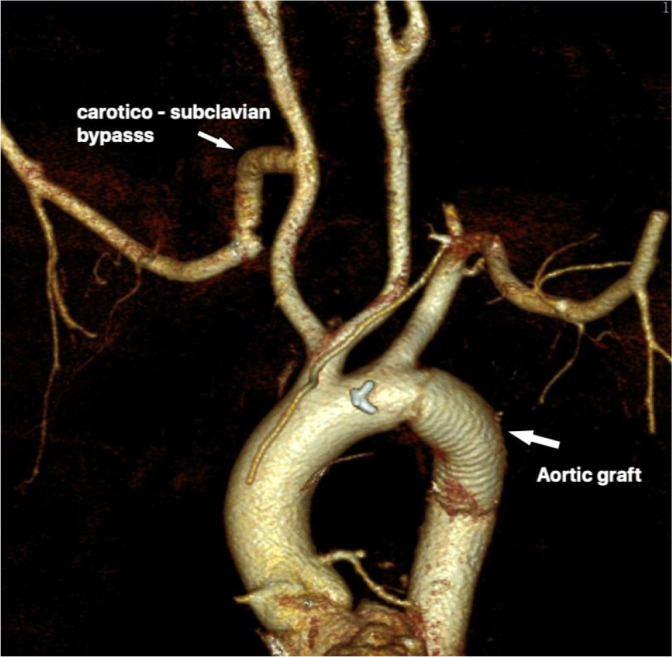
Postoperative control three-dimensional reconstructed computed tomography angiography of open aberrant right subclavian artery resection/ligation and right carotid-subclavian artery bypass.

**Fig. 3:**
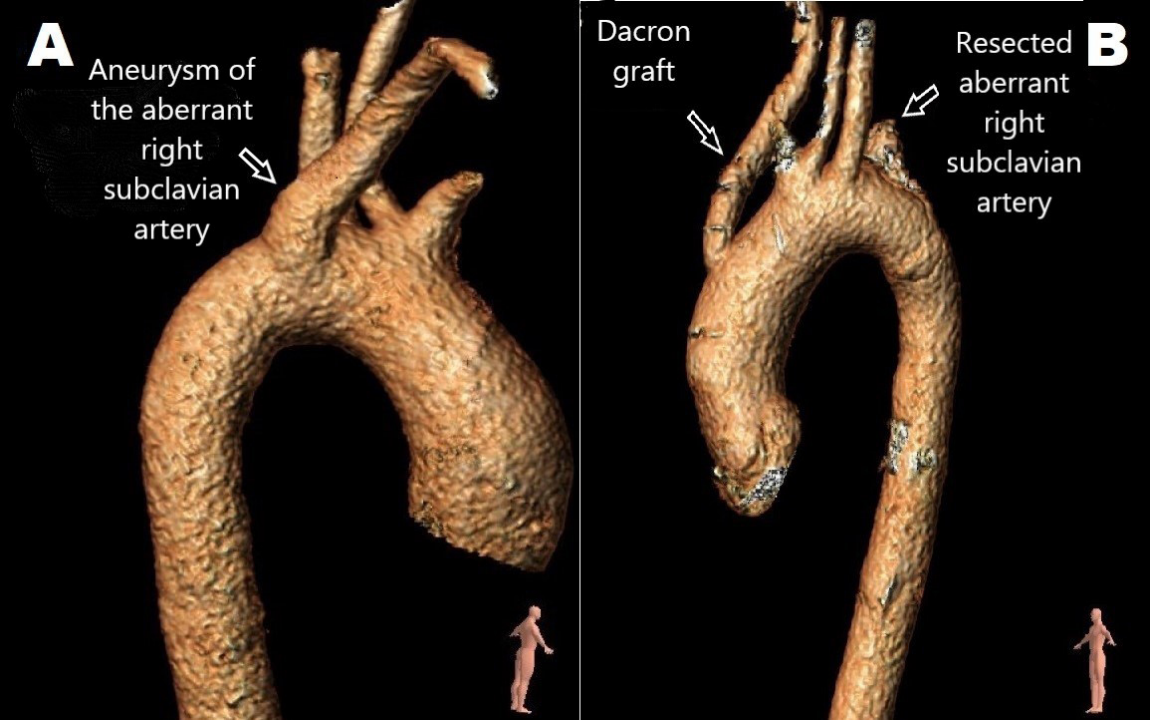
Preoperative (A) and postoperative (B) control three-dimensional reconstructed computed tomography angiography of open aberrant right subclavian artery resection/ligation and ascending aorta-right subclavian artery bypass.

Dysphagia symptom was present in all our patients as an indication for surgical correction; in addition, three of our patients had dyspnea. Aneurysm was present in six patients; one of the aneurysmatic ARSAs required emergency surgical intervention due to dissection. Hybrid procedure was applied to the first four patients by performing bilateral carotid-subclavian artery bypass (CScBp) and thoracic endovascular aortic repair (TEVAR) (Figure 1). In the first session, patients underwent bilateral CScBp operation. In the second session, TEVAR procedure was applied to the patients, and ARSA occlusion was achieved. In the second group of patients, both right CscBp operation and thoracic open aortic repair (TOAR) were performed in the same session (Figure 2). In the third group of patients, aortic graft operation was performed from the ascending aorta to the right carotid bypass and TOAR in the same session (Figure 3).

## RESULTS

Median age of the 11 patients (7 females/4 males) was 55 years (range 49 - 62). The treatment was performed with open surgery in seven patients (63.7%) and hybrid approach in four patients (36.3%). The demographic data of the patient groups who underwent three different approaches were analyzed, and no significant difference was found between them. ARSA was passing from the dorsal mediastinum to the esophagus in all 11 patients. All patients were symptomatic (dysphagia n=8 and dysphagia + dyspnea n=3). ARSA itself had aneurysmal dilatation in six patients (50%).

No patient had ruptured KD, but one had dissection of the ARSA (patient #5). Due to the sudden onset of symptoms, this patient underwent emergency operation. All other patients underwent elective surgery. Aneurysmatic ARSA was present in three of four patients who underwent bilateral CScBp + TEVAR. Four patients had dysphagia; one patient had dyspnea in addition to dysphagia. As a result of the two-stage operation, persisting esophageal compression was observed in two (50%) patients. Esophageal dilatation was applied to these patients, and symptoms were relieved. No endoleak was detected in the control CTA examinations performed in these patients who underwent the hybrid procedure.

All three patients who underwent right CScBp + TOAR had dysphagia. One patient in this patient group underwent emergency surgery due to dissection. No complications developed, and relief was observed in symptoms. Brachial plexus injury developed in one of the patients in this group. Right upper extremity weakness was observed in the postoperative period.

Of the four patients who underwent ascending aorta-subclavian artery bypass (AsScBp) + TOAR operation, four had dyspnea and two had dysphagia + dyspnea. These patients underwent elective anatomic bypass from the ascending aorta to the right subclavian artery and TOAR. In the postoperative period, pleural effusion developed in one patient, and relief was achieved only with thoracentesis. Hoarseness due to recurrent laryngeal nerve paralysis developed in one patient, and full recovery was observed with medical treatment at the three-month follow-up.

## DISCUSSION

ARSA is the most common anomaly involving the aortic arch with an incidence of 0.5 to 2.5%^[9]^. It originates after the left subclavian artery as the last branch of the aortic arch, turns from the back, and passes to the right. Although rare, it sometimes passes between the esophagus and the trachea or in front of the trachea. It is known that most of ARSA patients are asymptomatic. Symptomatic patients may present with different complaints. Dysphagia, dyspnea, or pneumonia complaints may occur due to esophageal or tracheal compression^[10]^. Symptoms may become more pronounced and worsen with advancing age. Although rare, complications such as aneurysmal dilatation, right arm ischemia, rupture, and fistulization can be seen^[11]^.

In the diagnostic sense, CTA and magnetic resonance angiography examinations are very important in revealing the anatomy of the anomaly and in the treatment strategy. Awareness of this anomaly is of clinical importance, as it may be associated with tracheoesophageal compression symptoms, aneurysm formation, or aneurysm rupture. It causes the appearance of an upper mediastina mass in both lesions. Recognition of ARSA is surgically important. In these cases, it is necessary to know the recurrent nerve tracing well. In addition, inadvertent placement of the cross-clamp proximal to the left subclavian artery during surgery may cause serious cerebral pathologies.

The first successful surgical treatment of ARSA was described by Gross in 1946^[12]^. In 1965, Bailey et al.^[12]^ reanastomosed ARSA from the right thoracotomy to the ascending aorta. Orvald et al.^[13]^ anastomosed the right subclavian artery to the right common carotid artery with only a cervical incision. Hybrid methods can also be applied in symptomatic ARSA patients in the light of developing technology and current approaches. We also used the hybrid approach in the first four patients in our published series. However, there was no regression in dysphagia complaints due to postoperative compression findings. Whereas, Vucemilo et al.^[14]^ reported that although no shrinkage in the aneurysm was observed in patients with hybrid treatment, symptomatic ARSA and compression findings were reduced. The low number of cases is thought provoking as to whether TEVAR has any symptom-relieving effect. Morris et al.^[15]^ used an Amplatzer Vascular Plug (St. Jude Medical, Saint Paul, Minnesota, United States of America) to occlude the proximal part of the abnormal right subclavian artery in a hybrid study. They then performed a CScBp via a supraclavicular incision. In such hybrid methods, dysphagia may be permanent or may be a risk factor for arterioesophageal fistula.

Apart from hybrid methods, there are also studies on open surgical applications. Van Son et al.^[16]^ surgically approached the mobilization of ARSA through a right thoracotomy, separating the vessel at its origin without leaving a long stump. Since the results of the hybrid method were not satisfactory in our clinical series, surgical repair methods were used. We performed CScBp + TOAR on three of our patients after our hybrid method applications. Among the postoperative results of the surgical technique we applied, we observed brachial plexus injury in one of these three patients. Despite the medical treatment, the postoperative long-term complication continued. In order to reduce the complication rates, we switched to the AsScBp + TOAR operation strategy, which is an anatomical bypass, in subsequent cases. Recurrent laryngeal nerve paralysis complication was observed in one of these four patients in the postoperative period. As a result, the patient developed hoarseness. Keiffer et al.^[17]^ emphasized the anatomical variations associated with ARSA in their study. These include abnormal origin of the right vertebral artery from the aorta or right common carotid artery, presence of a common carotid trunk, right-sided thoracic duct, and a non-recurrent laryngeal nerve.

When the laryngeal nerve is not recurrent, it originates from the vagus nerve in the neck and directly innervates the larynx. Although this anomaly is less important than others for the surgical treatment of ARSA, it is important to recognize it in patients who may require a carotid artery or thyroid procedure. The surgeon should identify and protect the vagus and recurrent laryngeal nerves while performing vascular exploration to avoid any neurological complication in the postoperative period. In posterior mediastina exploration, dissection close to the vessel may reduce the risk of neural damage. We think that left thoracotomy reduces the risk of laryngeal nerve injury in patients without aneurysm, especially the risk of left recurrent nerve injury near the source of ARSA.

Treatment indications and timing for ARSA is also a subject of discussion. Austin and Wolfe^[18]^ reported fatal rupture cases in 19% of the patients, and some of them showed dissection association. Therefore, many authors support fairly early interventions for these cases. In the case series that we have presented, we had to undergo an emergency operation with the development of dissection in an aneurysm in one of our patients. Complications that may occur if it is delayed may be mortal. Patient-specific characteristics, age, comorbid factors, anatomy, and potential risks of the procedure should be considered in determining the treatment strategy. Open repair may be more challenging and riskier in clinically challenging cases.

There is no standard treatment selection algorithm in current treatment methods and in this retrospective study. It is very difficult to compare hybrid or other surgical methods with each other. This work we have done can contribute to the decision-making process.

### Limitations

As our study was retrospective, there was no standard algorithm for choosing the treatment modality. Comparisons between the open and hybrid groups can be difficult. The small number of cases and the study’s retrospective nature are the most important limitations.

## CONCLUSION

Surgical treatment of symptomatic ARSA patients is a very challenging operation. Different surgeons on this subject apply different surgical methods, and there is no common consensus. With the developing technology, hybrid methods or combined surgical strategies can be applied. However, the benefits and harms of all of them are still among the controversial issues. In this study, we also presented three different surgical approaches, one of which is hybrid, and their results.

As a result of the comparison of these three techniques that we have applied in our clinic, we think that the AsScBp + TOAR approach is more appropriate because it has anatomical bypass, less risk of complications, and more successful surgical results. More case series are needed to further clarify this issue.
